# Digital Medicine System in Veterans With Severe Mental Illness: Feasibility and Acceptability Study

**DOI:** 10.2196/34893

**Published:** 2022-12-22

**Authors:** Sarah Gonzales, Olaoluwa O Okusaga, J Corey Reuteman-Fowler, Megan M Oakes, Jamie N Brown, Scott Moore, Allison A Lewinski, Cristin Rodriguez, Norma Moncayo, Valerie A Smith, Shauna Malone, Justine List, Raymond Y Cho, Amy S Jeffreys, Hayden B Bosworth

**Affiliations:** 1 Durham Center of Innovation to Accelerate Discovery and Practice Transformation Durham Veterans Affairs Medical Center Durham, NC United States; 2 Department of Population Health Sciences Duke University School of Medicine Durham, NC United States; 3 Mental Health Care Line Michael E. DeBakey Veterans Affairs Medical Center Houston, TX United States; 4 Department of Psychiatry and Behavioral Health Sciences Baylor College of Medicine Houston, TX United States; 5 Global Clinical Development Otsuka Pharmaceutical Development and Commercialization Inc. Princeton, NJ United States; 6 Pharmacy Service Durham Veterans Affairs Health Care System Durham, NC United States; 7 Durham Veterans Affairs Medical Center Durham, NC United States; 8 Department of Psychiatry and Behavioral Sciences Duke University School of Medicine Durham, NC United States; 9 School of Nursing Duke University Durham, NC United States; 10 Division of General Internal Medicine Department of Medicine Duke University Durham, NC United States

**Keywords:** ABILIFY MYCITE, digital medicine, adherence, aripiprazole, Veterans, qualitative methods, mental illness, mental health, medication, mobile phone

## Abstract

**Background:**

Suboptimal medication adherence is a significant problem for patients with serious mental illness. Measuring medication adherence through subjective and objective measures can be challenging, time-consuming, and inaccurate.

**Objective:**

The primary purpose of this feasibility and acceptability study was to evaluate the impact of a digital medicine system (DMS) among Veterans (patients) with serious mental illness as compared with treatment as usual (TAU) on medication adherence.

**Methods:**

This open-label, 2-site, provider-randomized trial assessed aripiprazole refill adherence in Veterans with schizophrenia, schizoaffective disorder, bipolar disorder, or major depressive disorder. We randomized 26 providers such that their patients either received TAU or DMS for a period of 90 days. Semistructured interviews with patients and providers were used to examine the feasibility and acceptability of using the DMS.

**Results:**

We enrolled 46 patients across 2 Veterans Health Administration sites: 21 (46%) in DMS and 25 (54%) in TAU. There was no difference in the proportion of days covered by medication refill over 3 and 6 months (0.82, SD 0.24 and 0.75, SD 0.26 in DMS vs 0.86, SD 0.19 and 0.82, SD 0.21 in TAU, respectively). The DMS arm had 0.85 (SD 0.20) proportion of days covered during the period they were engaged with the DMS (mean 144, SD 100 days). Interviews with patients (n=14) and providers (n=5) elicited themes salient to using the DMS. Patient findings described the positive impact of the DMS on medication adherence, challenges with the DMS patch connectivity and skin irritation, and challenges with the DMS app that affected overall use. Providers described an overall interest in using a DMS as an objective measure to support medication adherence in their patients. However, providers described challenges with the DMS dashboard and integrating DMS data into their workflow, which decreased the usability of the DMS for providers.

**Conclusions:**

There was no observed difference in refill rates. Among those who engaged in the DMS arm, the proportion of days covered by refills were relatively high (mean 0.85, SD 0.20). The qualitative analyses highlighted areas for further refinement of the DMS.

**Trial Registration:**

ClinicalTrials.gov NCT03881449; https://clinicaltrials.gov/ct2/show/NCT03881449

## Introduction

### Background

Suboptimal medication adherence is a significant problem for patients with serious mental illness (SMI), including those with schizophrenia, bipolar disorder, posttraumatic stress disorder, and major depressive disorder. Suboptimal adherence among these individuals may lead to symptom exacerbation, relapse, and hospital readmissions [1]. Moreover, suboptimal adherence to prescribed psychotropic medication is associated with increased mortality [1,2]. However, measuring medication adherence in the clinical setting is challenging and primarily subjective (ie, patient self-report) [3,4]. While objective methods such as pill counts and pharmacy refill data are helpful, they are time-consuming to calculate and often do not provide an accurate assessment of actual medication ingestion [5].

A digital medicine system (DMS), consisting of a drug-device combination, is a way to obtain objective treatment adherence data. DMS may enable patients with SMI to measure and report ingestion of atypical antipsychotic medications, most of which have broad therapeutic indications [6]. The collection of objective real-time data using a DMS enables providers to address nonadherence to medications, as well as facilitate interactions among patients and providers to promote and support medication adherence. Thus, adherence data obtained from a DMS may assist in understanding potential barriers to improving outcomes and more informed shared decision-making regarding a patient’s treatment for individuals with SMI [6].

### The ABILIFY MYCITE System

The ABILIFY MYCITE System is an example of a DMS developed to track adherence to oral aripiprazole, an atypical antipsychotic. This system enables patients and their mental health providers the opportunity to view real-time adherence data. Specifically, the ABILIFY MYCITE System is a drug-device combination product (aripiprazole tablets with a sensor) that comprises 4 separate components that enable the monitoring of treatment adherence by a patient and the patient’s provider (Table 1). The four components include (1) the ABILIFY MYCITE tablet (DMS tablet), an aripiprazole tablet embedded with an ingestible event marker sensor; (2) MYCITE System patch (DMS patch); (3) MYCITE System smartphone app (DMS app); and (4) MYCITE System dashboard (DMS dashboard).

**Figure 1:**
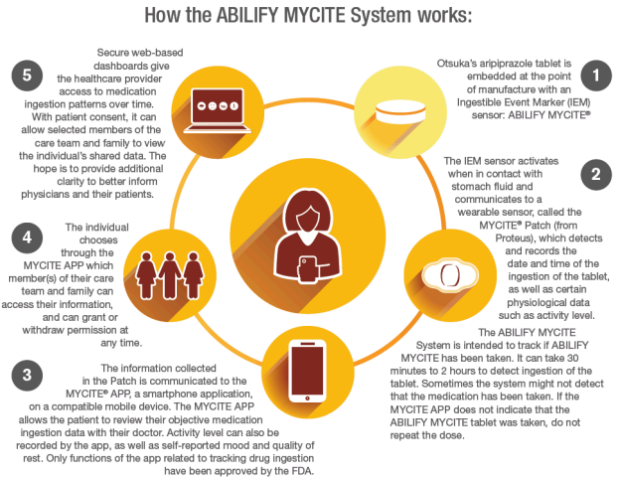
Schematic representation of digital medicine intervention. FDA: Food and Drug Administration; IEM: ingestible event marker. Image used with permission from Otsuka Development and Commercialization Inc.

Once a participant swallows the tablet, the ingestible sensor transmits an electrical signal that is detected and then recorded by software within the patch that is worn by the participant on the left rib cage. Using Bluetooth, the patch then transmits the aripiprazole ingestion data to the participant’s smartphone, which is then saved to the secure, cloud-based MYCITE System dashboard. Participants have the ability to view this medication data each day on their smartphone, while the patient’s provider, study team, and selected caregivers are able to view the data on the MYCITE System dashboard via the cloud-based server (Figure 1).

**Table 1:** Description of the 4 components of the digital medicine system (DMS)—Abilify MYCITE System.

Components	Description
ABILIFY MYCITE tablet (DMS tablet)	Aripiprazole tablet embedded with an ingestible event marker sensor
MYCITE System patch (DMS patch)	Wearable sensor patch that detects the signal from the ingestible event marker sensor after ingestion and transmits data to a smartphone
MYCITE System app (DMS app)	Smartphone app used to display medication ingestion information for the patient
MYCITE System dashboard (DMS dashboard)	Two separate web-based portals, one for health care providers and one for family and friends who care for the patient

### Purpose

Minimal research exists on the real-world comparison between a DMS and treatment as usual (TAU) with regard to medication adherence for individuals with SMI. Therefore, before testing a DMS in a large randomized controlled trial, we wanted to examine the feasibility and acceptability of a DMS among patients with SMI. As the Veterans Health Administration (VHA) is the largest health care provider for individuals with SMI in the United States [7,8], it provides a conducive setting for a clinical trial comparing DMS and TAU. Thus, the primary purpose of this study was to evaluate the impact of a DMS among Veterans (patients) with SMI as compared with TAU on medication adherence. The secondary purpose was to obtain patient and provider perspectives on the feasibility and acceptability of using a specific DMS, the ABILIFY MYCITE System. Notably, we use the abbreviation DMS for the remainder of the manuscript to represent the overall ABILIFY MYCITE System; however, we refer to specific components of the DMS (eg, tablet, patch, app, or dashboard) as needed.

## Methods

### Study Design

This was an open-label, 2-site, provider-randomized, prospective, 2-arm (DMS vs TAU) clinical trial. Patients assigned to the intervention group by provider randomization were enrolled in the DMS for a period of 90 days with the option to continue use for up to 9 additional months. Participants assigned to the control arm (TAU) continued to receive care as recommended by their mental health provider, which included their continued use of aripiprazole. Study duration for both groups was up to 12 months or study closeout, whichever came first. At the study conclusion, we used a descriptive qualitative analysis design and rapid qualitative analysis procedures to examine the feasibility and acceptability of using the DMS. All study procedures were approved by both sites’ respective institutional review boards and research and development committees. The trial is registered at ClinicalTrials.gov (NCT03881449), and the sponsor requested for the study to be concluded prematurely.

### Setting and Participants

Study participants were recruited from 2 VHA Medical Centers in Durham, North Carolina, and Houston, Texas. Eligible patients were aged ≥18 years and met the Diagnostic and Statistical Manual of Mental Disorders, Fifth Edition, criteria for schizophrenia, schizoaffective disorder, bipolar I disorder, or major depressive disorder. Additional study eligibility included (1) an active prescription for oral aripiprazole and (2) approval to participate in the study from their mental health provider. Exclusion criteria included (1) a current neurocognitive disorder that would affect the patient’s ability to complete the trial (eg, dementia); (2) the patient’s mental health provider determining that the patient was not fit to participate; (3) the patient being currently enrolled in an investigational drug trial, a medication management study, or program or participation in an investigational drug trial 30 days before trial enrollment; (4) the patient being pregnant, planning on becoming pregnant during the trial, or breastfeeding; (5) the patient failing an initial cognitive screener; (6) the patient having a known allergy to adhesive tape or any pertinent components of the DMS patch; (7) the patient not having skin on the anterior chest just above the lower edge of the rib cage, having dermatologic conditions, such as dermatitis or open wounds, in the location where the patch would be placed, or unwilling to refrain from the use of topical products on the skin patch sites; and (8) the patient having <20% proportion of days covered (PDC) with aripiprazole in the 6 months before enrollment.

### Screening and Recruitment

**Figure 2:**
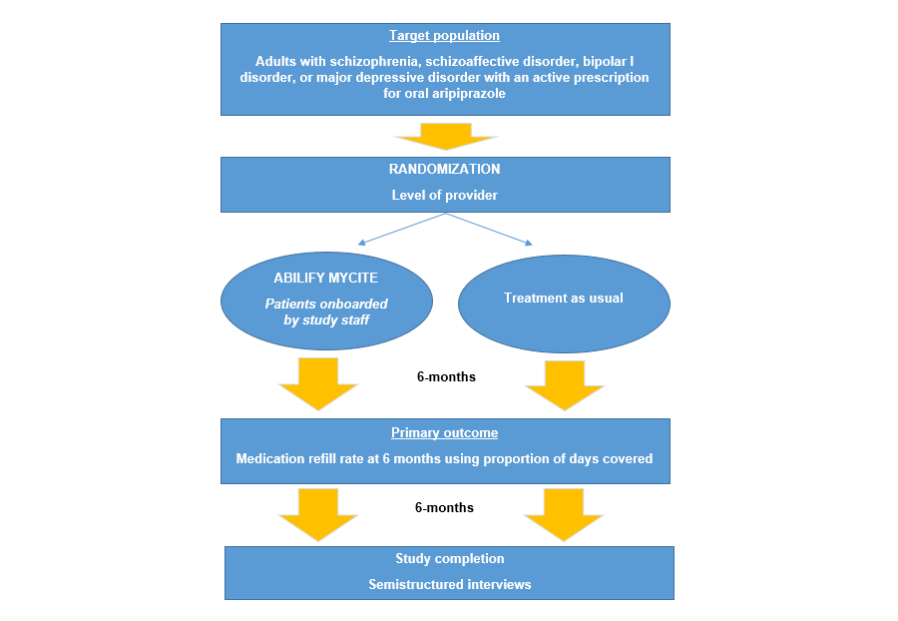
Schematic of enrollment procedures.

Potential participants were identified initially by a data pull from the electronic medical record. From this data pull, only patients of those providers who agreed to be involved in the study were screened further**.** Qualifying patients were sent an introductory letter in mail, describing the study and inviting them to contact the study team for more information and further eligibility screening by telephone. The study used an opt-out recruitment strategy that entailed contacting participants approximately 7 to 10 business days after a recruitment letter was sent, unless participants contacted the study team to indicate that they were not interested in participating. Once patients were confirmed eligible and were interested in participating, they were seen at a scheduled in-person baseline appointment. At the baseline study visit, written informed consent was obtained, smartphone compatibility was verified or a study-owned smartphone was provided if needed, and baseline assessments and surveys were completed (Figure 2)*.*

### Randomization

This was a provider-randomized clinical trial. Providers who agreed to have their patients approached and potentially enrolled were randomized in a 1:1 ratio using stratified block randomization. Provider randomization was stratified by site.

### Treatment Arms

#### DMS Arm

Once participants were enrolled in the trial, they were onboarded by a trained study team member. The onboarding process included obtaining DMS tablets from the site’s pharmacy, successfully placing the DMS patch on the skin at the proper location, and pairing the patch with the DMS app. The participant was then provided with additional training materials and contact information for the study team and the DMS product’s call center. Participants were encouraged to reach out to the company’s call center (DMS support) for technical assistance related to the DMS.

As part of the 12-month trial, participants in the DMS arm used the DMS tablet for 90 days. Participants then decided whether to continue beyond the initial 90 days. If participants discontinued the DMS tablet, they restarted their oral aripiprazole as prescribed by their provider. We continued to follow these individuals and obtained pharmacy refill data. Follow-up visits for both the DMS and TAU arms occurred at 3, 6, and 12 months. Early termination visits were completed for DMS arm participants only, per the study sponsor.

#### TAU Arm

Participants randomized to the TAU arm continued to receive usual care as provided by their mental health provider. Participants assigned to the TAU arm completed all required study visits and data collection surveys.

### Measures

#### Quantitative Measures

All study measures were collected by trained study staff at both sites.

##### Demographic and Clinical Data

A research assistant collected demographic data (eg, race, sex, age, and comorbidities); clinical data (eg, clinical diagnoses and medications); and data regarding the use of mobile health devices. Diagnoses were obtained from medical records and the following International Classification of Diseases codes were used: F33.0 (major depressive disorder), F31.0 (bipolar I disorder), F25.0 (schizoaffective disorder), and F20.0 (schizophrenia).

##### Medication Refill Adherence

The primary outcome was medication refill based upon the number of days covered from baseline to 6 months. Using 2 approaches, we measured adherence using PDC [9], a leading method used to calculate medication adherence at a population level. The first set of PDCs were calculated for 3 and 6 months independent of whether an individual was recommended to stop using the DMS (intention to treat). The second PDC measure was calculated as the number of days covered until there was documentation that a patient was recommended to stop using DMS or until the patient reported a problem with the intervention (eg, skin irritation) for the DMS group.

#### Qualitative Measures

Guided by rapid qualitative analysis procedures [10], we completed semistructured interviews to examine the feasibility and acceptability of the DMS to support medication adherence. We used a convenience sampling plan to identify participants enrolled in the DMS arm up to their 12-month participation in the study or the end of study activities, whichever came first. Providers whose patients were enrolled in the DMS arm were invited to complete an interview up to the date of the end of study activities. Research assistants contacted intervention patients currently enrolled in the study and invited participants and providers to complete interviews.

Interview questions inquired about the feasibility of and facilitators of and barriers to the DMS. Questions for the patients focused on medication experience (pre-enrollment), onboarding to DMS, system usability, satisfaction with support, and feedback. Questions for the providers inquired about the prescriber experience (prestudy), DMS dashboard account setup, system usability, satisfaction with support, and feedback. We used probes (eg, “Please describe your experience in greater detail” and “What do you mean?”) to obtain greater detail and clarify responses. Interviews were completed by a trained research assistant and included a notetaker who recorded responses via a structured note form. After the interview, the research assistant and notetaker debriefed and reviewed interview responses in the context of other interviews. Interviews were conducted via the telephone and were recorded but not transcribed. Patient and provider interviews lasted for 43 (SD 12) minutes on average.

### Analytic Strategy

#### Quantitative Measures

Oral aripiprazole refill was measured by the number of days covered from baseline to 3 and 6 months using PDC in both intention-to-treat and DMS-engaged analyses. Owing to the small number of participants, we conducted descriptive analyses rather than a model-based approach. The intention-to-treat analyses used all data from baseline to 3 or 6 months, depending on the outcome, while the DMS-engaged analysis censored participants in the DMS arm at system discontinuation.

#### Qualitative Measures

We followed rapid analysis procedures for data analysis and Microsoft Excel (version 2002) to support coding and analysis. Two authors (AAL and SG) reviewed all notes and debrief notes taken during interviews with patients and providers. These authors used thematic analysis [11] and the matrix method [12] to analyze and identify salient themes across all interviews. We established rigor and validity by independently coding and summarizing all data, discussing emerging codes and thematic groupings during meetings, and reviewing findings with the larger study team.

### Ethics Approval

This study was approved by the Durham Veterans Affairs Medical Center Institutional Review Board (ID number 02188) on January 19, 2019.

## Results

### Sample

**Figure 3:**
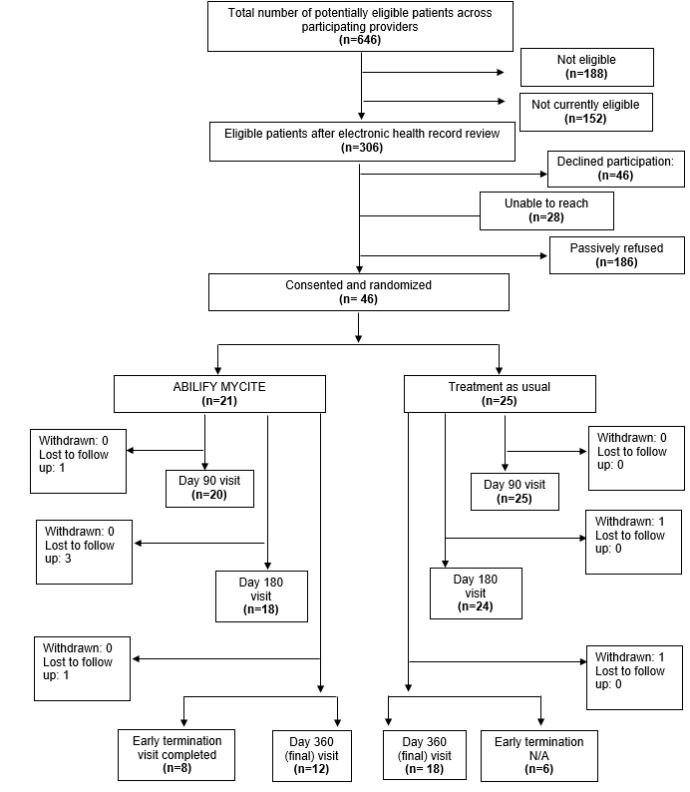
Enrollment. N/A: not applicable.

A total of 26 providers were randomized for this trial (Durham: 22/26, 85%; Houston: 4/26, 15%). Of the eligible patients from participating providers, a total of 46 patients consented and enrolled in the trial (Durham: 28/46, 61%; Houston: 18/46, 39%), with 21 (46%) participating in the DMS arm and 25 (54%) participating in the TAU arm (Figure 3)*.* We issued 6 study-owned smartphones to patients to facilitate enrollment in the study. The sample was on average aged 53 (SD 13.3) years and mostly male (33/46, 72%), 52% (24/46) self-reported as Black, and 15% (7/46) had a high school education or less. The clinical diagnoses breakdown for enrolled patients included 54% (25/46) with major depressive disorder, 7% (3/46) with schizophrenia, 11% (5/46) with schizoaffective disorder, and 28% (13/46) with bipolar I disorder. Before study enrollment, half (23/46, 50%) of the enrolled patients had downloaded a health app onto their mobile phone, and approximately one-third (14/46, 30%) of the participants had used a wearable tech device such as a fitness tracker or smartwatch (Table 2)*.*

**Table 2:** Participant demographics (N=46).

			DMS^a^ (n=21)		TAU^b^ (n=25)
Age (years), mean (SD)			54.67 (12.73)		51.64 (13.40)
**Sex, n (%)**					
	Male	17 (81)		16 (64)	
	Female	4 (19)		9 (36)	
	Intersex	0 (0)		0 (0)	
**Race, n (%)**					
	Black	12 (57)		12 (48)	
	White	8 (38)		13 (52)	
	Asian	1 (5)		0 (0)	
**Education, n (%)**					
	High school or less	5 (24)		2 (8)	
	Any college	14 (67)		21 (84)	
	Graduate school	2 (10)		2 (8)	
**Clinical diagnosis, n (%)**					
	Major depressive disorder	14 (67)		11 (44)	
	Bipolar I disorder	3 (14)		10 (40)	
	Schizoaffective disorder	2 (10)		3 (12)	
	Schizophrenia	2 (10)		1 (4)	
**Prestudy mobile device use, n (%)**					
	Health app	9 (43)		14 (56)	
	Wearable technology	8 (38)		6 (24)	

^a^DMS: digital medicine system.

^b^TAU: treatment as usual.

### Quantitative Outcomes

In the intention-to-treat analyses, PDC over 3 and 6 months was 0.82 (SD 0.24) and 0.75 (SD 0.26) in the DMS arm and 0.86 (SD 0.19) and 0.82 (SD 0.21) in the TAU arm, respectively. In the DMS-engaged analysis, the DMS arm had 0.85 (SD 0.20) PDC over the period (Table 3)*.*

Patients in the DMS arm stayed engaged with the DMS for 144 (SD 100; median 147; range 0-376) days on average. Among the participants in the DMS arm, 5 (24%) stopped using the system by 3 months because of skin irritation adverse events from the use of the DMS patch.

**Table 3:** Proportion of days covered (PDC) for the drug of interest.

PDC	DMS^a^ (n=21), mean (SD)	TAU^b^ (n=25), mean (SD)
3 months	0.82 (0.24)	0.86 (0.19)
6 months	0.75 (0.26)	0.82 (0.21)
DMS engaged	0.85 (0.20)	N/A^c^

^a^DMS: digital medicine system.

^b^TAU: treatment as usual.

^c^N/A: not applicable.

### Qualitative Outcomes

#### Overview

Qualitative interviews were completed with 14 patients and 4 providers, with half (7/14, 50% and 2/4, 50%, respectively) of each group from each of the 2 sites. Patient respondents were mostly male 86% (12/14), while 64% (9/14) were Black, 29% (4/14) were White, and 7% (1/14) were Asian. Overall, 86% (12/14) of patient respondents used the DMS for ≥90 days; 43% (6/14) used the DMS for ≥180 days. Of the providers, 50% (2/4) identified as male, 75% (3/4) were providers, and 25% (1/4) were prescribing pharmacist.

We identified 5 themes when analyzing the patient data and 5 themes for the providers’ data that described the feasibility and acceptability of using the DMS. Patient themes included pre-enrollment adherence strategies and interest in the DMS, positive impact on medication adherence, system usability challenges, support needs, and suggested improvements to system design and functionality. Provider themes included prestudy concerns for patient medication adherence and interest in the DMS, concerns with the DMS (prestudy), DMS dashboard usability issues and support, challenges in impact of the DMS, and suggestions to increase provider use.

#### Semistructured Interviews—Patient Responses

##### Pre-enrollment Strategies and Interest in the DMS

Patients described the following pre-enrollment medication adherence strategies: maintaining a routine and setting up reminders through environmental cues (eg, seeing pills on their counter, a reminder from their spouse, or from reminders on their phone or calendar). Regarding their pre-enrollment medication adherence strategy, a patient stated that the “hardest part for me is sometimes I can’t remember if I took my meds that day, so that’s why I try to take them first thing in the morning when I wake up...” Patients were interested in using the DMS because they felt that the DMS would help them with their medication adherence. A patient shared the following statement:

...I have issues with taking medication on time, not remembering to take it, skipping doses, so the way that the [DMS] would remind you...that would be helpful to someone.

Many patients expressed interest in the technology used in the DMS, including a patient who stated the following:

I was interested because of the new technology...having the [DMS] app so you can see what your daily activities are.

##### Positive Impact on Medication Adherence

Patients reported the DMS made them more mindful of taking their medication. For example, a patient shared the following statement:

[The DMS] made me more cognizant of what time my dosages were and definitely to make sure that I took [my medications] daily...when I opened my phone, I would see the DMS app and remember, oh, I have to take my medicine in the morning.

The visual reminder of seeing their DMS patch and the DMS app on their phone acted as a prompt to take their medication. In addition, the DMS app’s medication notifications to log missed doses into the DMS app reinforced the habit of taking their medication consistently. A patient stated the following:

The [DMS app] itself, it asks you a series of questions why you forgot to take it and so when you’re going through that it just feels like it’s telling you to not forget it again. I definitely thought it was helpful...it reinforced that good habit of remembering to take your medication every day.

##### System Usability Challenges

Many patients shared that they experienced problems with DMS patch connectivity and skin irritation. A patient described the challenges they experienced as follows:

If the [DMS] patch wasn’t paired and I wasn’t paying attention to the [DMS] app, I would take my medication and it wouldn’t register and that was very frustrating...I [also] had problems with the DMS patch adhering and when it wouldn’t adhere, it would unpair

with the DMS app

A patient who experienced skin irritation from the DMS patch stated the following:

It seemed the more that I changed the DMS patch, the more irritated my skin got with the DMS patch...no matter where I put it.

Patients also expressed that the DMS patch was uncomfortable to wear, as the DMS patch caught on things when working in tight spaces and fell off when the patient sweated because of weather or physical activity.

Patients experienced challenges with the DMS app during DMS tablet registration (eg, DMS app would freeze and would need to be rebooted, technical assistance needed, or unable to log missed doses). A patient stated the following:

I had a lot of issues with the [DMS] app also, the [DMS] app was freezing up on me and I would have to call [DMS support] and we would have to walk through it, we’d have to uninstall it and reinstall it to get the [DMS] app to not be frozen anymore so that it would download the [DMS] patch and pair the [DMS] patch.

Overall, despite assistance from study staff, patients felt the DMS was complicated to learn because of the numerous steps needed to complete each process, and many patients shared that they discontinued use because of recurring challenges.

##### Support Needs

Patients expressed that their onboarding to the DMS was helpful because of the in-person, one-on-one training with their local study staff members. Patients who called DMS support felt that DMS support was professional and knowledgeable in describing step-by-step solutions. However, patients reported that DMS support was not always able to resolve the patient’s issues and provide the patient with a long-term fix for their challenges with using the DMS. A patient described this as follows:

I continued to have the same issue and that’s why I discontinued using the DMS...[DMS support]’s recommendation to change the patch and re-pair it wasn’t a long-term fix.

Patients preferred in-person support for resolving issues with the DMS because study staff members could see their smartphone and DMS app in real time. When asked about support for the DMS, a patient stated the following:

Since the study team was local, I was more inclined to call them if I needed help with something...just being there and having them explain to me face-to-face and answer my questions right away, it was helpful...and also that they were able to see my DMS app and see what was going on.

##### Suggested Improvements to System Design and Functionality

Patient feedback for the DMS included two main suggestions: (1) developing a smaller DMS patch or an alternative way to track ingestion that did not involve wearing a DMS patch and (2) improving the usability and functionality of the DMS app as well as the reliability and accuracy of the DMS as a whole.

Regarding patch improvement suggestions, a female patient shared the following:

If I could have placed the DMS patch somewhere else on my body, I might have continued...if it could be worn somewhere discreetly on the body that would be ideal...where it had to be at the top of my torso, that was problematic for me...I don’t think it would be problematic for a man or someone with a smaller chest.

Several patients described how the DMS patch was cumbersome to wear, wearing the DMS patch for long periods led to skin irritation, or that they experienced issues with the DMS patch sticking to their skin. Another patient shared the following:

I did not like the DMS patch...it gave me a rash...if we could figure out some other way of doing it without the DMS patch involved that would be wonderful.

A patient who experienced DMS patch adherence issues stated the following:

I think having to have it on your torso is a problem...how active I am, it just couldn’t stay on me. Maybe if it was moved to an extremity...even if it was a fitness tracker, that would have made that part of the whole process much easier.

Regarding suggested improvements to the DMS app usability and functionality, a patient said the following:

When you log into the DMS app, it logs you off rather quickly...the login information wasn’t saved, so it was time consuming to access the DMS app.

In addition, another patient suggested the following:

If your old DMS patch doesn’t upload information [to the DMS app]...having a mechanism to bypass that and record it and send it to DMS support, so you can continue going through the pairing process with the new DMS patch

would be helpful

Finally, some patients suggested that DMS support be more proactive (eg, calling patients periodically to check in rather than patients needing to call DMS support to obtain technical assistance). A patient stated the following:

For people like us that’s not as savvy with technology as others are...if [DMS support] have called me and checked, maybe [using the DMS] would have been easier and simpler.

In addition, patients described an interest in receiving further training and material on strategies for addressing potential challenges to the DMS, including technical issues with the DMS app, the DMS, DMS patch connectivity, and DMS patch contact. A patient expressed that they would have liked to receive written material, as this patient wanted to refer back to information during the study.

#### Semistructured Interviews—Provider Responses

##### Prestudy Concerns for Patient Medication Adherence and Interest in the DMS

Providers shared a common concern for patient compliance with medication and acknowledged several challenges to medication adherence (eg, disease specific, health care system related, and side effects of medications). A provider explained, “It’s hard to know, by patient report, how consistently they’ve been taking the medication...” A provider described challenges patients have in refilling medications as follows:

It’s very daunting to get the refills and if the refills are done, they often times...get them late or they forget to order it...it’s not like they don’t want to intentionally not take their medicine.

Overall, providers were interested in the DMS, as this system could provide an objective measure of adherence instead of self-report. A provider verbalized as follows:

I’m always looking for ways to help my patients...take ownership of their own care and to improve quality of care. Those are the two things that drew me to the DMS.

Providers also shared that a common issue can be reconciling what a patient says compared with medication data in the electronic health record. A provider said the following:

I was drawn to something like technology that would help both on the provider’s end and the patient’s end to overcome that kind of barrier.

##### Concerns with the DMS (Prestudy)

Providers expressed concerns with the implementation of DMS in their patient populations, specifically those who experience paranoia and would be apprehensive of using the DMS to track medication adherence. A provider said the following:

My initial reaction was paranoid, psychotic patients might be a bit concerned that they’re taking a pill that has a sensor...I was thinking that they might not be willing to take such a medication where one would know [provider or family member] whether they are taking it or not.

An additional concern was around the use of the DMS patch because of the provider’s experience with skin sensitivity to adhesives.

##### DMS Dashboard Usability

Providers experienced frustration with the multistep process to log into the DMS dashboard via the notification email. This frustration led them to not check notification emails or log into the DMS dashboard. Improvement suggestions included streamlining the log-in process for the DMS dashboard (eg, the ability to save password and not needing to re-enter it each time) and including additional details (eg, missed dose or multiple doses taken at one time) in the notification email to encourage them to log into the DMS dashboard more frequently. Notably, none of the providers interviewed called DMS support for assistance with the DMS dashboard, rather these providers called the study staff because of their accessibility.

##### Challenges in the Impact of the DMS

Providers stated that the objective medication adherence data for each patient in the DMS was helpful. However, owing to numerous usability challenges (eg, data inaccuracy and multistep log-in process), these providers did not use the DMS dashboard, rather they relied on the study team for information on their patient’s medication data.

##### Suggestions to Increase Provider Use

Provider feedback for DMS centered on (1) improving accessibility to the DMS dashboard and (2) change in DMS data management to improve workflow. Providers suggested a streamlined process for receiving notifications such as including additional information in the notification email and embedding the DMS log-in into the electronic health record (eg, an embedded link taking them to the DMS dashboard) to make it easier to integrate into their regular workflow. A provider stated the following:

If there was some way we could incorporate [ingestion data] in our templated notes...while we are writing, documenting the notes, we could click the link to the DMS dashboard...that might work.

Finally, many providers recommended that the DMS dashboard and data should instead be managed by an individual in the clinic (eg, nurse or clinical coordinator), as this individual can summarize patient data for the provider. A provider said the following:

...If the DMS could be created in a way that there will be a go-between, an intermediary between the provider and the patient on the DMS, who would be monitoring, more closely, the ingestion data...[and summarize] this is number of days of adherence, number of days of not adhering...so that the prescriber has that information right in front of them, even before seeing the patient.

## Discussion

### Principal Findings

This open-label, 2-site, 12-month, provider-randomized trial assessed aripiprazole refill adherence in patients with SMI and examined patient and provider perspectives on the feasibility and acceptability of DMS for this population in the VHA health care system. Our study showed that there was no notable difference in the refill rates between the DMS and TAU arms in intention-to-treat analyses. Among the users of the DMS, the PDC by refill rates were 0.85 (SD 0.20) for those patients when engaged in the DMS. Qualitative findings indicated several challenges to the feasibility and acceptability of the DMS for patients and providers. These challenges included technical issues and contact issues with the DMS patch, including skin irritation and adherence on the skin, and affected the length of user participation as well as overall confidence and interest in using the DMS as a reliable and accurate method of tracking medication ingestion. Both patients and providers discussed recommendations to improve the patient-provider experience and overall satisfaction with the DMS. Notably, patients and providers commented on how to increase confidence in using the DMS for patients with SMI, particularly with patients who may not have extensive experience with smartphones, Bluetooth technology, and health app use.

Our results indicated extended patient use of the DMS to manage their SMI. However, our results should be interpreted with caution, as the DMS did not appear to outperform TAU in this study, and enrollment was only a fraction of our original goal. Patients were asked to use the DMS for 90 days, at which time they, along with their provider, could decide whether to continue using the DMS beyond 90 days and up to the full 12 months of study participation. Compared with previous studies using a DMS with an ingestible sensor pill for a period of 8 [13] or 12 [14] weeks, the use of the DMS lasted up to 12 months, which provided insight into DMS use that was 90 days or longer (12/21, 57%), with 29% (6/21) of respondents opting to use the DMS for 180 days or more.

In another trial using the same DMS [13] as in our study, adherence was measured by the proportion of days with good DMS patch coverage (ingestible event marker registration). However, in our study, medication adherence was assessed by the PDC on aripiprazole using pharmacy record data. Owing to the prevalence of patient-reported technical difficulties with DMS tablet registration for the DMS and provider concern for data accuracy that was described during the qualitative interviews, PDC was a preferred measure of medication adherence in this study. Compared with a medication adherence rate of ≥80% reported over an 8-week period (when good DMS patch coverage was reported) in the previous study [13], our study saw 82% adherence in our intention-to-treat analysis over a 3-month (approximately 12 weeks) period, which was comparable. However, this rate changed to 75% at 6 months in our study, which reveals an area of further study into the potential cause of lower adherence rates after 90 days.

Our study’s qualitative findings highlighted concerns regarding DMS patch use and contact issues (skin irritation and adherence to skin). In a recent study that evaluated patient responses to using a comparable digital medicine program with an ingestible sensor coencapsulated with antiretroviral therapy medication, patients reported similar issues as seen in our qualitative findings, including issues with patch adherence to skin and overall frustration with using the digital medicine program patch [15]. As detailed earlier and in our findings, DMS patch issues affected the length of user participation; consideration should be given to making improvements to the usability of the DMS patch, particularly long-term use, to improve the patient experience and adherence to the DMS.

### Limitations

Several considerations should be acknowledged. First, enrollment and data collection for the study ended early per the study sponsor’s request, presumably before more null or potentially negative findings could emerge. Second, technology issues may have affected the continued use of the DMS and engagement in the intervention. Third, a limitation for consideration with regard to adherence is the potential of the Hawthorne effect related to the observation of both participant groups (DMS and TAU) in a trial. This may have affected adherence rates as measured by PDC.

In addition, while qualitative interviews were conducted with 14 VHA patients who used the DMS during their study participation, only 4 providers were interviewed. Overall, provider engagement with the DMS dashboard was limited—some providers were unable to share feedback on their experience with the DMS dashboard and the impact that the use of the DMS had on their patients and on patient-provider communication. Providers who consented to participate in the qualitative interviews shared their level of interaction with the DMS dashboard, and interview questions were designed to capture potential barriers to use and to gather feedback on how to increase provider use. Individuals enrolled had a relatively high rate of refill at baseline; future studies using DMS may want to focus on individuals who are having greater challenges with refill adherence. Finally, some patients were interviewed about their DMS experience several months or sometimes up to 1 year after enrollment, which could have affected how well the patients remembered specific details about their experience.

### Strengths

Despite the aforementioned limitations, we collected data on the impact of DMS use in a specific patient population, while also gathering detailed feedback on patient and provider experiences, and suggested modifications and considerations for DMS improvements. Evaluation of this novel DMS in the VHA health care system provided insight into real-world use for increased use in community-based, private practice, and public health care settings.

### Conclusions

Our study of a DMS in patients with SMI did not demonstrate a detectable improvement in medication adherence. Our findings indicate critical issues to consider in improving the feasibility and acceptability of a DMS for patients and providers. Specifically, our study highlights the importance of conducting a feasibility and acceptability trial and collecting quantitative and qualitative data to further refine and improve the DMS for long-term adherence.

## Abbreviations

DMS: digital medicine system
PDC: proportion of days covered
SMI: serious mental illness
TAU: treatment as usual
VHA: Veterans Health Administration
